# Prospects for Biocontrol of *Vibrio parahaemolyticus* Contamination in Blue Mussels (*Mytilus edulus*)—A Year-Long Study

**DOI:** 10.3389/fmicb.2018.01043

**Published:** 2018-06-05

**Authors:** Bukola A. Onarinde, Ronald A. Dixon

**Affiliations:** School of Life Sciences, University of Lincoln, Lincoln, United Kingdom

**Keywords:** *Vibrio parahaemolyticus*, temperate estuary, chromogenic agar, *Mytilus edulis*, RAPD-PCR, bacteriophage, genetic diversity

## Abstract

*Vibrio parahaemolyticus* is an environmental organism normally found in subtropical estuarine environments which can cause seafood-related human infections. Clinical disease is associated with diagnostic presence of *tdh* and/or *trh* virulence genes and identification of these genes in our preliminary isolates from retail shellfish prompted a year-long surveillance of isolates from a temperate estuary in the north of England. The microbial and environmental analysis of 117 samples of mussels, seawater or sediment showed the presence of *V. parahaemolyticus* from mussels (100%) at all time-points throughout the year including the colder months although they were only recovered from 94.9% of seawater and 92.3% of sediment samples. Throughout the surveillance, 96 isolates were subjected to specific PCR for virulence genes and none tested positive for either. The common understanding that consuming poorly cooked mussels only represents a risk of infection during summer vacations therefore is challenged. Further investigations with *V. parahaemolyticus* using RAPD-PCR cluster analysis showed a genetically diverse population. There was no distinct clustering for “environmental” or “clinical” reference strains although a wide variability and heterogeneity agreed with other reports. Continued surveillance of isolates to allay public health risks are justified since geographical distribution and composition of *V. parahaemolyticus* varies with Future Ocean warming and the potential of environmental strains to acquire virulence genes from pathogenic isolates. The prospects for intervention by phage-mediated biocontrol to reduce or eradicate *V. parahaemolyticus* in mussels was also investigated. Bacteriophages isolated from enriched samples collected from the river Humber were assessed for their ability to inhibit the growth of *V. parahaemolyticus* strains *in-vitro* and *in-vivo* (with live mussels). *V. parahaemolyticus* were significantly reduced *in-vitro*, by an average of 1 log−2 log units and *in-vivo*, significant reduction of the organisms in mussels occurred in three replicate experimental tank set ups with a “phage cocktail” containing 12 different phages. Our perspective biocontrol study suggests that a cocktail of specific phages targeted against strains of *V. parahaemolyticus* provides good evidence in an experimental setting of the valuable potential of phage as a decontamination agent in natural or industrial mussel processing (343w).

## Introduction

*V. parahaemolyticus* is a Gram-negative bacterium commonly found in marine and estuarine coastal environments (Feldhusen, 2000; Ceccarelli et al., 2013; Letchumanan et al., 2014; Malcolm et al., 2015; Raghunath, 2015) and can cause shellfish-related gastroenteritis (Hazen et al., 2015; Raghunath, 2015), wound infection and septicaemia in sub-tropical environments (Daniels et al., 2000; Zhang and Orth, 2013). Gastrointestinal infections in humans cause vomiting, diarrhea, abdominal pain, nausea, fever, chills with an onset time of 2–48 h after consumption. This organism is frequently isolated from a range of raw seafoods, such as crab, fish, lobster, oyster, shellfish, and shrimp (Wang et al., 2015; Letchumanan et al., 2016) and mussels. *V. parahaemolyticus* multiplies in the human intestinal tract and produces one or more toxins that contribute to the symptoms. Clinical infections are reported from diverse serovars, but since not all isolates are pathogenic to humans, the ubiquitous distribution of *V. parahaemolyticus* in the environment poses a challenging problem for predictive diagnosis and control. To monitor the prevalence of *V. parahaemolyticus* from environmental sources, organisms need to be efficiently identified and differentiated from other Gram-negative bacteria that might be present. Traditional isolation techniques consisting of Thiosulphate Citrate Bile Salt Sucrose (TCBS) agar has caused difficulties distinguishing *V. parahaemolyticus* from other vibrio species in environmental samples. Although this traditional selective media is based on sugar fermentation, it is still widely used for the isolation of *Vibrio* species from natural and clinical environments (Blanco-Abad et al., 2009) but a chromogenic agar containing substrates for β-galactosidase has been recognized as an improvement for the detection of *V. parahaemolyticus* in marine samples. In addition, *V. parahaemolyticus* can be identified by the detection of haemolysin genes—all strains display a species-specific haemolysin coded by a thermolabile (*tlh*) gene. In clinical cases, two well-described haemolysins, thermostable direct haemolysin genes (*tdh*) and *tdh*—related haemolysin (*trh*) are present (Honda and Iida, 1993; Nishibuchi and Kaper, 1995; Bej et al., 1999; Harth et al., 2009; Johnson et al., 2012; Zhang and Orth, 2013; Letchumanan et al., 2014; Raghunath, 2015). The detection of these gene sequences by PCR amplification targeted by specific oligonucleotide primers are the most important predictive measure of the pathogenicity of *V. parahaemolyticus* strains for human illness (Johnson et al., 2012). Reliable molecular methods have been developed for the sub-species typing of *V. parahaemolyticus* (Wong and Lin, 2001; Khan et al., 2002; Bilung et al., 2005) and RAPD-PCR analysis has commonly been used for the study of genetic relationships in *Vibrio* species (Tada et al., 1992; Bilung et al., 2005; Gonzalez-Escalona et al., 2005; Ellingsen et al., 2008). The method generates “fingerprints” that can be used to compare bacteria both at the inter-species and intra-species level with high discriminating power and (a) does not require previous knowledge of sequences in the DNA of the isolate under study (b) produces a DNA pattern that allows comparison of many loci simultaneously, (c) simple and relatively low cost and requires only nanogram amounts of template DNA. Previous studies employing RAPD-PCR have analyzed *Vibrio* communities isolated from many parts of the world (Sudheesh et al., 2002; Domingue et al., 2003; Gonzalez-Escalona et al., 2005) although only Ellingsen et al. (2008) documents RAPD-PCR analysis of *V. parahaemolyticus* environmental isolates in northern Europe.

The distribution and composition of potentially pathogenic *V. parahaemolyticus* in the environment requires careful monitoring as ocean warming may well change the geographical distribution in the coastal estuaries (Baker-Austin et al., 2013). With changes in patterns of global warming, human infections with *V. parahaemolyticus* traditionally confined to warm subtropical geographical areas may represent increased risks developing in temperate regions (McLaughlin et al., 2005). Simple and clear methodology is required to monitor temperate regions for isolates acquiring virulence genes which would represent increased risks for the community. The control and management of many pathogens faces ever increasing challenges for healthcare and food environments due to the increasing rates of antibiotic resistance. The antimicrobial susceptibility of *V. parahaemolyticus* isolates in particular has changed during the past few decades with diminished sensitivity to antimicrobials (Daramola et al., 2009). Public and professional concerns about the reduction of treatment options in healthcare have triggered global efforts to find novel alternatives to antibiotics, such as bacteriophages (Caplin, 2009). Although bacteriophage therapeutics have been recognized and used for many years in the early to mid-twentieth century their popularity declined rapidly in the West with the introduction of antibiotics. In Poland, between 1983 and 1987, Slopek et al. published a series of research articles on the effectiveness of phages against human infections caused by several bacterial pathogens, including multidrug-resistant mutants, (Slopek et al., 1983a,b, 1984, 1985a,b,c, 1987). Additional studies in Tbilisi, Georgia during 1963 and 1964 (Babalova et al., 1968) reported strong evidence of the effectiveness of phages on *Shigella* in children having gastrointestinal disorders. More recently there has been a reawakening of interest in “phage therapy” for both human and animal applications (Parisien et al., 2008) revealing that “phage therapy” is effective in clinical situations and virtually free of serious complications (Fortuna et al., 2008). The potential to treat infections in food livestock (Atterbury et al., 2007; Johnson et al., 2008), plants and aquaculture (McIntyre et al., 2007) has been demonstrated at least at a research level with good examples in luminous vibriosis caused by *V. harvey* in poultry (Vinod et al., 2005), and *Aeromonas hydrophila* in fish (Wu et al., 1981). Phage interventions are particularly attractive as a potentially useful strategy for the decontamination of live animals during the processing or harvesting stages of the product since phages are considered a GRAS product and have been approved by the FDA (Bren, 2007) and unlike antibiotics they appear not to demonstrate any known negative pharmacological effects (Parasion et al., 2014). More robust control of *V. parahaemolyticus* by bacteriophage as an intervention could therefore be useful in the harvesting of mussels and oysters from polluted beds in aquaculture. Bacteriophage strategies for bacterial pathogen decontamination have the advantages of being self-perpetuating, highly discriminatory, safe, natural, and cost-effective. Some of the drawbacks with their use is that there is theoretical potential for the transduction of undesirable characteristics from one bacterial strain to another. Although the significance of transduced resistance is yet to be determined the limited host range and potential emergence of phage resistant bacterial mutants may or may not be important in real life situations (Wilton, 2014).

In this study, bacteriophages specific to *V. parahaemolyticus* from estuarine water, sediments, and mussels will be identified, their host range and their ability to reduce genetically diverse *V. parahaemolyticus* isolates tested *in vitro*. The effects of an experimental cocktail of bacteriophage on *V. parahaemolyticus in vivo* in harvested mussels in the laboratory will be investigated.

## Materials and methods

### Retail supply

Raw shellfish samples (oysters, whelks) for our preliminary study were purchased from a local market that sources shellfish products from Grimsby, a large fishing town in Lincolnshire situated on the south bank of the Humber estuary.

### Sampling site

**Figure 1:**
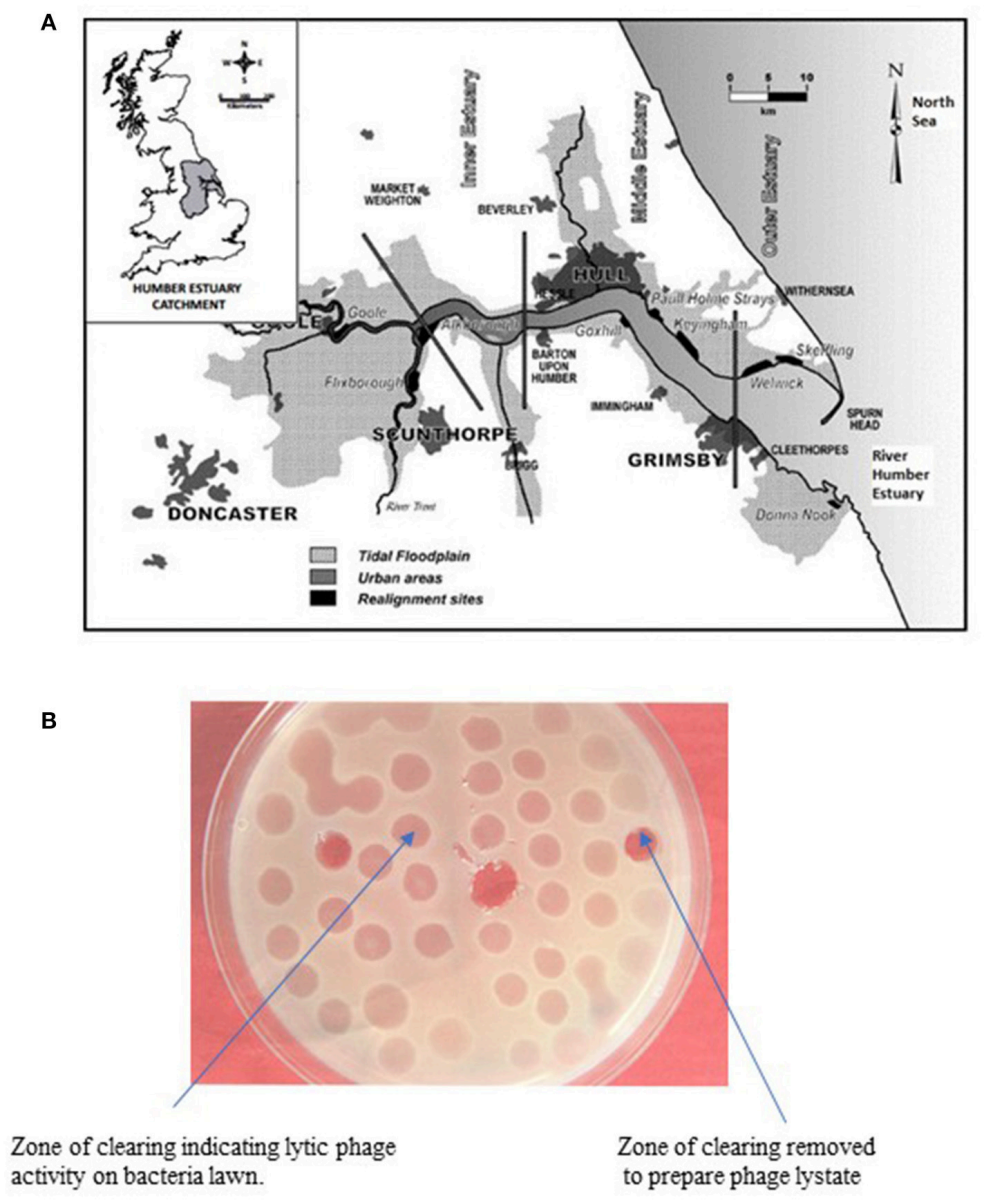
**(A)** Map of the East Coast Region of England showing sampling site (Cleethorpes) along the River Humber. **(B)** Representative agar plate showing lytic activity of bacteriophage on lawn of host *V. parahaemolyticus*.

The surveillance study was conducted in Grimsby (Cleethorpes an adjacent resort) on the East coast of England (Figure 1A) situated at the mouth of the Humber estuary on the North Sea. The Humber estuary is one of the largest estuarine sites in Britain at ~9.5 km from “Spurn Point” which is its recognized entrance and 6.5 km wide at its mouth (GPS coordinates; Latitude: 53° 33′ 23.99″ N; Longitude: 0° 01′ 21.60″ E). Grimsby has one of the most important fish docks in Europe and there are over 70 shellfish harvesting point along the River Humber and more than 6 seaside recreation resorts.

### Sample collection

For the isolation of *V. parahaemolyticus*, a total of 117 environmental samples were collected over 39 weeks between September and August and analyzed. Samples collected included shellfish (blue mussels; *Mytilus edulis*) (*n* = 39), seawater (*n* = 39), and sediment (*n* = 39). For each month between the study period (with exception of the month of June), samples were collected at least twice a month and up to 4 and 5 times per month when this was possible. Samples were collected around noon time when there was low tide. Collection of mussels was achieved at low tide by hand pulling mussels from natural clusters formed on the pillars of the pier, collected samples were then placed directly in a sterile polyethylene bag. Surface water samples were collected directly into a sterile plastic container while surface sediment samples were scooped using a sterile glass container and placed in another sterile glass jar. All samples were transported to the laboratory on ice in a portable insulated box. Samples were analyzed between 4 and 6 h of sample collection.

### Environmental parameters

During collection of samples, temperature and salinity were measured at the sampling site. Temperature (seawater and sediment) and salinity were measured using a portable digital thermometer and a portable calibrated WPA cm35 conductivity meter OHMS-1 (μmhos/cm) respectively.

### Culture media

Chromogenic vibrio (CV) agar (CHROMagar Co., France) and Thiosulphate Citrate Bile Salts Sucrose (TCBS) agar (Fluka, BioChemika, Switzerland) were used for the isolation of *V. parahaemolyticus* [formula: Agar: 15 g/l; Peptone: 8 g/l; Yeast extract: 8 g/l; Salts: 51.4 g/l; Chromogenic mix: 0.3 g/l; Final pH 9.0 ± 0.2 at 37°C].

### Sample preparation and isolation method for *V. parahaemolyticus*

In the laboratory, mussel samples were immediately removed from the polythene bag, washed, and scrubbed under running potable water to remove sediment, debris, and attached algae. Samples were then opened aseptically with a sterile knife and the mussel flesh and shell liquid were placed in a sterile jar. Isolation of *V. parahaemolyticus* was carried out by weighing 25 g of individual sample (extracted mussel flesh) into a sterile stomacher bag with 225 ml of Alkaline Peptone Water (APW) containing 2% NaCl. The seawater and sediment sample bag were mixed thoroughly by shaking the bag for ~1 min while the mussels sample was homogenized in a stomacher for 1 min. All samples were then incubated at 37°C for 6–8 h, for pre-enrichment and then individual samples were serially diluted in APW. Using the surface spread plate technique, 0.1 ml of the diluted sample was spread onto the CV and TCBS agar and incubated at 37°C for 18–24 h. After incubation violet and green colonies were recognized on CV and TCBS agars respectively as being typical of *V. parahaemolyticus* colonies. Three putative *V. parahaemolyticus* colonies were then randomly selected from a single plate for each sample analyzed. Individual colonies were suspended in Tryptone Soya Broth (TSB) supplemented with 3% sodium chloride, and incubated at 37°C overnight to obtain broth cultures of isolated bacteria.

### Statistical analysis—surveillance

The relationship between the counts of *V. parahaemolyticus* and environmental parameter (temperature and salinity) was analyzed by linear multiple regression. Samples without observable *V. parahaemolyticus* isolates were assigned the lower limits of detection for each sample. The limits of detection assigned for *V. parahaemolyticus* was 1 × 10^3^cfu per ml or g for seawater and sediment samples. The total *V. parahaemolyticus* counts were log-transformed (base 10) and analyzed by regression of the mean Log_10_ counts of duplicate samples against environmental factors (temperature and salinity). Statistical package Microsoft Excel 2013 and XLSTAT 2014 was used to perform all statistical analyses.

Table 2 provides a list of the pathogenic *V. parahaemolyticus* strains obtained from Chile and Spain and the reference strain used in this study.

### Molecular analysis

#### PCR assays

##### Specific *V. parahaemolyticus* genes

Bacterial DNA was prepared from overnight broth cultures of individual *V. parahaemolyticus* colonies grown in Luria Bertani (LB) broth supplemented with 3% NaCl and incubated at 37°C. Supernates containing bacterial template DNA were used (as previously described by Bej et al., 1999) directly in specific-PCR for the detection of *tlh* gene. The PCR assay was carried out in 0.5 ml Eppendorf tubes with reaction mixtures consisting of sterile PCR grade distilled water: Mercury™ brand Taq DNA 2.0X MasterMix, 6.0 μl DMSO: 2.0 μl; Primer Tlh-F−100 pmol/μl: 1.0 μl; Primer Tlh-R−100 pmol/μl: 1.0 μl, (Table 1A) (Bej et al., 1999); Template DNA−3.0 μl. The reaction mixture was subjected to 35 amplification cycles according to the protocol shown in Table 1B and PCR amplification products (10 μl) and 2 μl of 6x gel loading dye (NEB) were electrophoresed on 1.5% agarose gels at 100 V for 2 h. The gel was stained with ethidium bromide (0.5 μg/ml) and resolved bands visualized under UV transillumination with an imaging system (Kodak, Gel Logic 100).

**Table 1A:** Primers used for specific PCR targets.

**Primers**	**Sequence Genes (5′-3′)**	**Amplicon size**
Tlh-F	5′-AAA GCG GAT TAT GCA GAA GCA CTG-3′	450 bp
Tlh-R	5′-GCT ACT TTC TAG CAT TTT CTC TGC-3′	450 bp
Tdh-D3	5′-CCA CTA CCA CTC TCA TAT GC-3′	251 bp
Tdh-D5	5′-GGT ACT AAA TGG CTG ACA TC-3′	251 bp
Trh-R2	5′-GGC TCA AAA TGG TTA AGC G-3′	250 bp
Trh-R6	5′-CAT TTC CGC TCT CAT ATG C-3′	250 bp

**Table 1B:** Amplification conditions for specific-PCR and RAPD-PCR assay.

**Gene**	**Initial denaturation**	**Denaturation**	**Annealing**	**Extension**	**Cycles**
*tlh*	96°C /5 min	94°C /30 sec	55°C /30 secs	72°C /1 +7 min	35
*tdh* and *trh*	96°C /5 min	94°C /1 min	55°C /1 min	72°C /1 +7 min	35
RAPD-PCR	94°C /5 min	94°C /1 min	36°C /1 min	72°C /2 +5 min	45

##### Pathogenic *V. parahaemolyticus* genes

PCR was performed separately for *trh and tdh ge*nes on isolated *V. parahaemolyticus* strains and reference organisms (Table 2). The PCR assay was carried out in 0.5 ml Eppendorf tube with reaction mixtures according to the method of Tada et al. (1992). The primers and PCR conditions used are described in Tables 1A,B, PCR amplification products (10 μl) and 2 μl of 6x gel loading dye (NEB) were electrophoresed on 1.5% agarose gels at 100 V for 2 h. The gel was stained with ethidium bromide (0.5 μg/ml) and resolved bands visualized under UV transillumination using an imaging system (Kodak, Gel Logic 100).

**Table 2:** Clinical isolates of *V. parahaemolyticus* used in this study including 6 *V. parahaemolyticus* strains kindly provided by Prof. R.T. Espejo (Chile), 2 strains provided by Dr. Martinez-Urtaza (Spain) and reference strains.

**Strain name**	**Original strain name/number**	***tlh* gene**	***tdh* gene**	***trh* gene**
CHL1	HVC 275	+	+	+
CHL4	PMA 114	+	–	+
CHL5	VpKx(RIMD2210633)	+	+	–
CHL8	TAA 66	+	–	–
CHL9^*^	COA20	+	+	+
CHL10^*^	ATA 69	+	+	+
CHL14	Kx737	+	+	+
CEF7	V05/031	+	–	–
CEF8	V05/032	+	+	–
CEF16	VP05/425	+	–	–
SPA 2^*^	447/00	+	+	–
SPA3^*^	30824	+	+	–
SPA4	9808/1	+	+	–
*Vibrio parahaemolyticus*	NCTC 10903 (ATCC17802)	+	+	–

Our results of species-specific PCR targeting the tlh, tdh, and trh genes are shown

* *strains used for phage cocktail study; other strains used for RAPD analysis*.

#### RAPD-PCR assay

For the genetic diversity studies, genomic DNA was extracted from the organisms by the mini-preparation method described by Ausubel et al. (1987). Amplification reactions were performed in 25 μl volume in a 0.5 ml Eppendorf tubes. The reaction mixture contained 12.5 μl of Mercury™ *Taq* DNA polymerase master mix (NEB) 8.5 μl of PCR grade water, 1.0 μl of 100 pmol RAPD primer, 1.5 μl of DMSO, and 2.0 μl of template DNA. PCR amplifications were performed as shown in Table 1B. Two primers were chosen for the fingerprinting profile and cluster analysis. The 10-mer oligonucleotide primers were described in previous studies: Gen1-50-08 with oligonucleotide gene sequence 5′-GAG ATG ACG A-′3 (Bilung et al., 2005) and OPD-16 with sequence 5′-AGG GCG TAA G-′3 (Sudheesh et al., 2002). PCR amplification product (7 μl) and 2 μl of gel loading dye were loaded on to a 1.5% agarose gel and electrophoresed at 70 V for 3 h. The resolved bands were visualized under UV transillumination and images captured. Bands were read visually from “fingerprints” generated by the two primers and a data matrix was generated for each primer. A dendrogram was constructed using the data matrix of all the bacterial strains based on Unweighted Pair Group Method with Arithmetic means (UPGMA) using a GelCompar II (Applied Maths) software package.

### Determination of antibiotic susceptibility of host bacteria

Antibiotic sensitivity testing was performed by the Bauer-Kirby disk diffusion method (Bauer et al., 1966).

### Isolation of bacteriophages

Bacteriophages were isolated from seawater or mussels by various enrichment methods described previously by Baross et al. (1978). Briefly, seawater of equal volumes was added to *V. parahaemolyticus* cultures in log-phase grown in double strength TSB supplemented with 3% NaCl and incubated at 30°C overnight. For isolations from mussels, 25 g of homogenized flesh was added to 225 ml of tryptic soy broth (TSB) or phage buffer (1M Tris HCl, 0.1M NaCl, 8 mM MgSO4, 0.1 g/l gelatin (pH 7.5) and either shaken vigorously for 10 min or enriched at room temperature with slow aeration for 96 h. The mixtures were centrifuged at 13,000 × g for 15 min at 4°C and the supernatant filtered through a 0.45 μm filter. The filtrates (phage lysates) were stored at 4°C until required.

### Detection of lytic activity of phages on panel of *V. parahaemolyticus* reference strains and isolates

Phage lytic activity was detected by surface inoculation of 25 μl of the filtrate (phage lysates) onto previously prepared lawns of *V. parahaemolyticus* grown on tryptic soy agar (TSA) supplemented with 3% NaCl in a traditional plaque assay. After incubation at 30°C for 24 h, the presence of zones of clearing (clear or turbid) were measured and recorded (Figure 1B).

### Characterization of phage lysates

Areas of agar demonstrating clear zones were removed using a sterile pipette and each inoculated into 5 ml of phage broth (prepared by mixing Peptone: 15 g/l; Bacto-Tryptone: 8 g/l; Yeast extract: 1 g/l; NaCl: 25 g/l, pH adjusting to 7.2 with NaOH and autoclaved before aseptically adding 1 mM of MgSO_4_ and 1 mM of CaCl_2_.) containing an early log phase culture of *V. parahaemolyticus*. Following incubation at room temperature for 24 h, the contents was centrifuged at 12,000 × g for 15min at 4°C. Phage titers were determined by the traditional overlay method, i.e., diluted in phage buffer and plated using the soft-agar technique (Adams, 1959). The plates remained at room temperature for 24–48 h. Plaque diameter measurements and numbers were recorded.

### Bacteriophage DNA extraction

Phage lysate (500 μl) obtained from individual plaques was added to 100 μl of 0.5 mM EDTA in a sterile 1.5 ml micro-centrifuge tube. Following 30 min at room temperature 600 μl of water-saturated phenol was added and the tube gently mixed following centrifugation at 13,000 × g for 30 s and 300 μl of the aqueous phase was removed. The phage DNA was stored at 4°C until required.

### Restriction fragment length polymorphism-PCR (RFLP-PCR) analysis of phage DNA

To determine the diversity of phage isolates and the approximate genome sizes from the fragments generated, restriction digestion analyses were carried out with three restriction enzymes. The restriction enzymes *Hae*III, *Hind*III, and *EcoR*I (Shivu et al., 2007) were used according to the manufacturer's (NEB) instructions. A total volume of 15 μl reaction mixture which contained 13 μl of phage DNA 0.5 μl, *Hae*III, *Hin*dIII, or *Eco*RI, and 1.5 μl restriction enzyme buffer (NEB) were placed in 0.5 ml Eppendorf tube. The mixture was then incubated at 37°C for 2 h. The reaction product was subjected to agarose gel electrophoresis (0.8%) at 120 V for 1 h.

### Sodium dodecyl sulphate-polyacrylamide gel electrophoresis (SDS-PAGE) of phage protein

SDS-PAGE was performed according to the method of Laemmli (1970), using a separation gel of 12% acrylamide. Phage lysate (7–14 μl) were dissolved in (3–6 μl) SDS-PAGE sample buffer (containing 2-mercaptoethanol), mixture was then heated at 100°C for 5 min. Samples were then inoculated into SDS-PAGE gel and a protein ladder (NEB™) of size ranging from 6.5 to 212 kDa, was used as protein size marker. After electrophoresis the acrylamide gel was submerged in 20 ml of “instant blue” (a coomassie based blue staining solution) obtained from RunBlue™, Expedeon, for 1 h on an orbital shaker and then placed in the fridge overnight. The resulting protein band sizes were determined using GelCompar II software.

### Determination of the host range of isolated phage

A plaque assay method (Adams, 1959) was used to determine the ability of the phages to form individual plaques on one or more *V. parahaemolyticus* strains. Each phage was named after its respective host bacteria with addition of prefix Φ (phi).

### Preparation of VP10 phage cocktail

Equal volumes of each prepared phage lysate was centrifuged at 13,000 × g for 15 min. The resulting supernates were then filtered (0.45 μm filter) and stored at 4°C until required.

### *In-vitro* activity of bacteriophage

Overnight cultures (1 ml) of the selected *V. parahaemolyticus* were added to sterile 1.5 ml Eppendorf tubes and centrifuged at 13,000 × g for 5 min. The supernatant was removed and cell pellets from each strain re-suspended in 1 ml of bacteriophage lysate containing ~1.3 × 10^2^ pfu/ml in TSB supplemented with 3% NaCl. The control samples were also re-suspended in 1 ml of sterile TSB supplemented with 3% NaCl. After thorough mixing, tests and control tubes were incubated at temperatures within the temperature range (5–43°C) for growth of *V. parahaemolyticus* known to support the growth and multiplication of the organism in the laboratory. Test and control tubes were incubated for 24 h at 30°C, 37°C (optimal temperature) or 40°C for 24 h. Serial dilutions (in sterile distilled water) were prepared and plated onto TSA plates supplemented with 3% NaCl. Plates were incubated at 37°C for 24 h. Bacterial colonies were counted and results expressed in cfu/ml.

### *In-vivo* activity of bacteriophage

*In vivo* experiments were conducted in 2.5 l glass beakers positioned in a refrigerator at 4°C. Three duplicate containers containing 8–16 harvested mussels each were used together with equal volumes of seawater and sediment and the bacterial numbers of *V. parahaemolyticus* present in each was determined as cfu/ml (g), before the introduction of the phage cocktail. Ten milliliters of VP10 phage cocktail (~0.1 × 10^6^ pfu) was used. Controls containing mussels, sediment and seawater only in beakers were established. Every 24 h for 3 days samples were removed from treated and controls and 25 g of mussel homogenized prior to plating on to CV agar plates. Colonies were counted after incubation at 37°C for 24 h from both treated and control tanks. Variables such as temperature, salinity and pH were monitored and recorded throughout the period of study with a portable temperature probe and conductivity meter and calibrated pH meter respectively.

### Statistical analysis—laboratory analyses

Variations between treatments were analyzed using repeated measures analysis of variance (ANOVA) and Bonferroni adjustment in SPSS version 14.0. Analysis of variance (ANOVA) is a hypothesis-testing procedure used to determine if mean differences exist for two or more treatments. The purpose of ANOVA in this study is to find out whether the differences between the control and treatment samples are simply due to random error (sampling errors) or whether there are systematic treatment effects that have caused counts in one group to differ from the other. In addition to ANOVA Bonferroni *Post*- *de nova* test was used to determine where significant difference between each of the variables lie.

## Results

### Isolation of *V. parahaemolyticus* on selective agar

CV agar and Thiosulphate Citrate Bile Sucrose Salt (TCBS) were employed as primary isolation media. *V. parahaemolyticus* were identified as purple and green colonies on CV agar and TCBS respectively. The use of TCBS in differentiating between sucrose positive and negative strains of *V. parahaemolyticus* was found to be difficult, especially when attempting to detect *V. parahaemolyticus* within a mixed bacterial culture. The use of chromogenic agar was more effective in detecting *V. parahaemolyticus* isolates than TCBS. It was observed that more presumptive colonies of *V. parahaemolyticus* were detected on chromogenic agar than on TCBS, when the same amount of sample was plated onto the different selective agar.

### Bacterial confirmation and virulence test (PCR)

PCR amplification of the *tlh, tdh*, and *trh* genes yielded amplicons, which were ~450, 251, and 250 bp, respectively. In this present study, detection of these pathogenic markers revealed that 2 *V. parahaemolyticus* isolated from retail shellfish samples collected during the preliminary study possessed the *tdh* gene (see Table 2).

### Prevalence of *V. parahaemolyticus*

**Figure 2:**
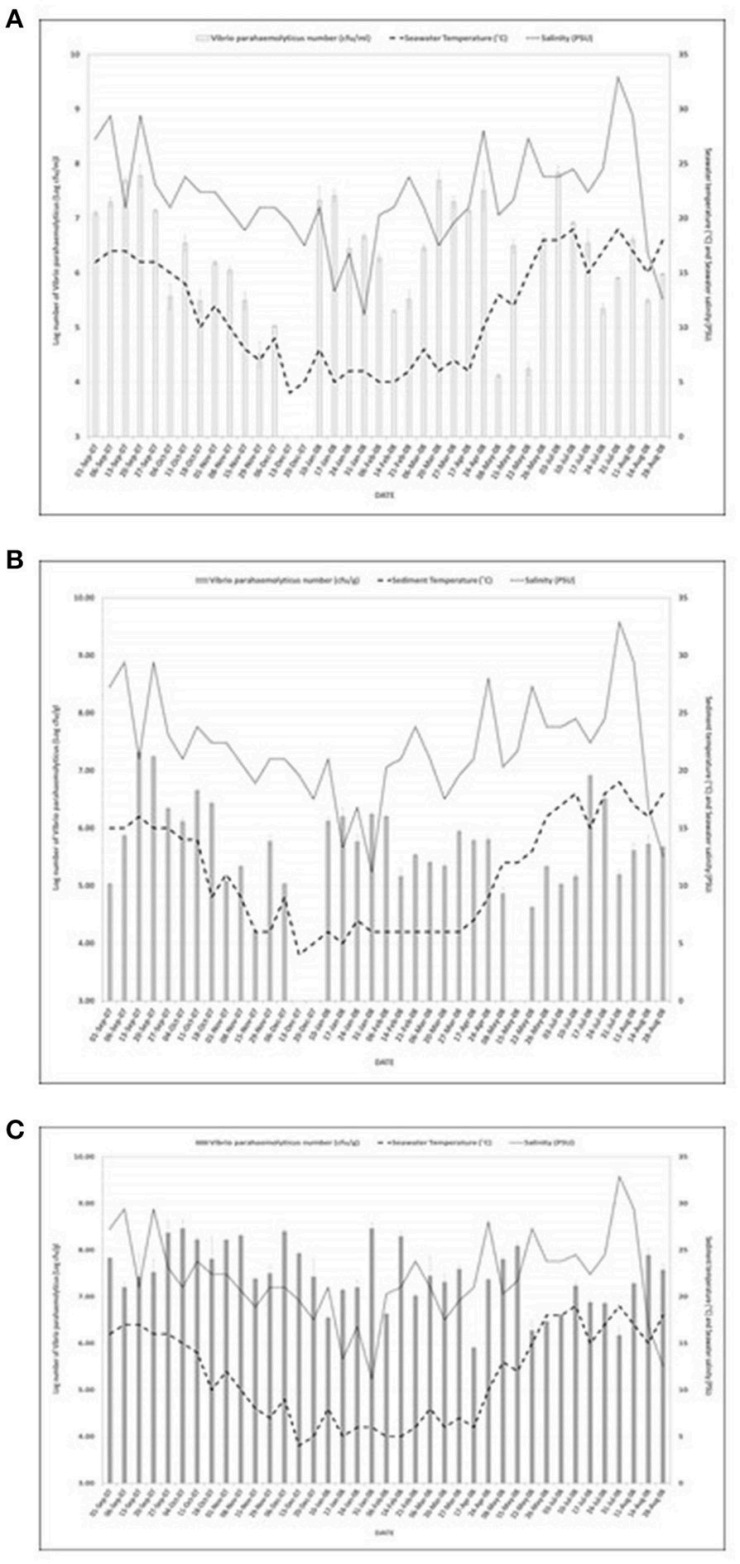
**(A)** Number of *Vibrio parahaemolyticus* isolated from **seawater** samples in relation to seawater temperature and salinity. *R*^2^ = 0.068; *P* = 0.262 (seawater) and *R*^2^ = 0.024; *P* = 0.153 (salinity). *R*^2^ = 0.066; *P* = 0.256 (temperature) and *R*^2^ = 0.006; *P* = 0.074 (salinity) were observed for counts of *V. parahaemolyticus* in sediment. And *R*^2^ = 0.014; *P* = −0.120 (temperature) and *R*^2^ = 0.094; *P* = −0.307 (salinity) were observed for counts of *V. parahaemolyticus*. **(B)** Number of *Vibrio parahaemolyticus* isolated from **sediment samples** in relation to sediment temperature and salinity. *R*^2^ = 0.068; *P* = 0.262 (seawater) and *R*^2^ = 0.024; *P* = 0.153 (salinity). *R*^2^ = 0.066; *P* = 0.256 (temperature) and *R*^2^ = 0.006; *P* = 0.074 (salinity) were observed for counts of *V. parahaemolyticus* in sediment. And *R*^2^ = 0.014; *P* = - 0.120 (temperature) and *R*^2^ = 0.094; *P* = −0.307 (salinity) were observed for counts of *V. parahaemolyticus*. **(C)** Number of *Vibrio parahaemolyticus* isolated from **mussels** in relation to seawater temperature and salinity. *R*^2^ = 0.068; *P* = 0.262 (seawater) and *R*^2^ = 0.024; *P* = 0.153 (salinity). *R*^2^ = 0.066; *P* = 0.256 (temperature) and *R*^2^ = 0.006; *P* = 0.074 (salinity) were observed for counts of *V. parahaemolyticus* in sediment. And *R*^2^ = 0.014; *P* = −0.120 (temperature) and *R*^2^ = 0.094; *P* = −0.307 (salinity) were observed for counts of *V. parahaemolyticus*.

Figure 1A shows the study location of the surveillance study and Figures 2A–C present *V. parahaemolyticus* counts in seawater, sediment and mussels respectively throughout the year. Isolation of *V. parahaemolyticus* was 100% (39/39) in mussel, 92.3% (36/39) in sediment, and 94.9% (37/39) in seawater. The organisms were detected in all mussel samples throughout the study period and the counts appeared to be independent of variation recorded in salinity and temperature. Linear regression analysis of the mean Log_10_ counts of *V. parahaemolyticus* (in seawater, sediment, and mussels) and environmental parameters showed no significant relationship (*P* < 0.05) with temperature or salinity. Correlation coefficient and significant level observed when counts of *V. parahaemolyticus* in seawater were compared with (a) change in seawater temperature were *R*^2^ = 0.068 and *P* = 0.262 respectively, (b) change in salinity were *R*^2^ = 0.024 and *P* = 0.153, respectively. Correlation coefficient and significant level observed when counts of *V. parahaemolyticus* in sediment were compared with (a) change in sediment temperature were *R*^2^ = 0.066 and *P* = 0.256, respectively, (b) change in salinity were *R*^2^ = 0.006 and *P* = 0.074, respectively. Correlation coefficient and significant level observed when counts of *V. parahaemolyticus* in mussels were compared with (a) change in seawater temperature were *R*^2^ = 0.014 and *P* = −0.120, respectively, (b) change in salinity were *R*^2^ = 0.094 and *P* = −0.307, respectively.

### RAPD-PCR assay

**Figure 3:**
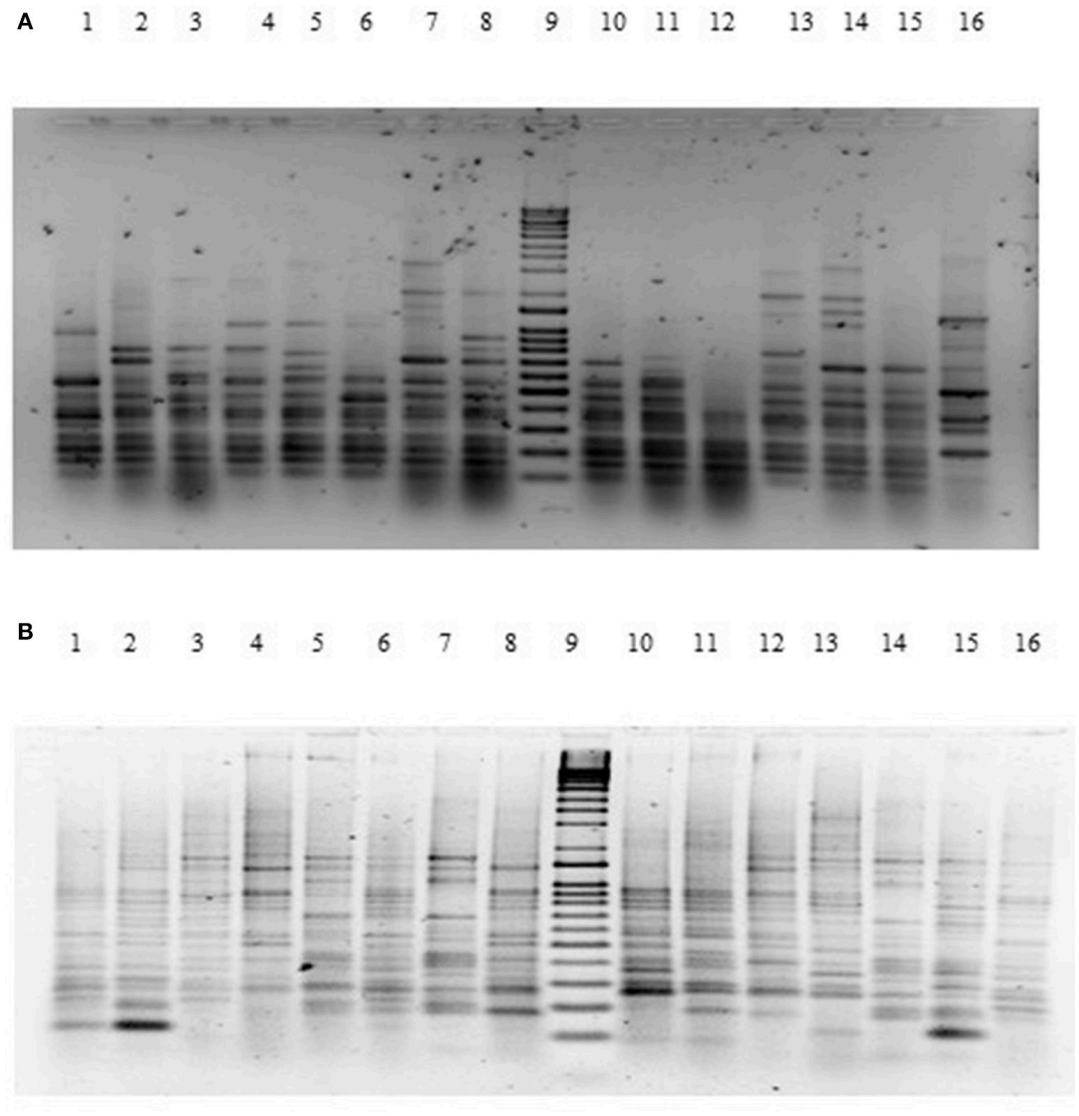
**(A)** Agarose (1.5%) gel electrophoresis of RAPD-PCR products for primer GEN1-50-08 of *V. parahaemolyticus* isolates. Lanes Lane 1: BD1; Lane 2: BD2; Lane 3: BD3; Lane 4: BD4; Lane 5: BD6; Lane 6: BD7; Lane 7: BD35; Lane 8: BD9; Lane 9: Q-4 DNA ladder; Lane 10: BD14; Lane 11: BD37; Lane 13: BD33; Lane 14: BD54; Lane 15: BD34; Lane 16: BD39. **(B)** Agarose (1.5%) gel electrophoresis of RAPD-PCR products for primer OPD16 of *V. parahaemolyticus* isolates. Lane 1: CHL16; Lane 2: CHL1; Lane 3: CHL14; Lane 4: BD7; Lane 5: BD84; Lane 6: BD1; Lane 7: BD3; Lane 8: BD5; Lane 9: Q-4 DNA ladder; Lane 10: BD34; Lane 11: BD30; Lane 12: BD29; Lane 13: BD77; Lane 14: BD71; Lane 15: BD47; Lane 16: BD75.

The genetic relatedness of the *V. parahaemolyticus* isolates studied was demonstrated using RAPD-PCR and revealed a high degree of heterogenicity between *V. parahaemolyticus* isolates recovered from the Humber and isolates from Chile, Spain, and other regions of the UK. Representative results of the RAPD patterns obtained are shown in Figures 3A,B as examples. GelCompar version 4.1 software was used to construct a phylogenetic dendrogram. The data from primers Gen1-05-08 and OPD-16 generated a distance matrix and *V. parahaemolyticus* isolates were allocated to 5 and 6 major clusters for each primer respectively (Figures 3A,B).

### Isolation and characterization of bacteriophages

The phage lysate from water or mussel samples after enrichment produced clear zones of lysis on bacterial lawns as shown in Figure 1B. A total of 61 bacteriophages producing distinctive plaque sizes were recovered from the Humber and both turbid and clear plaques were observed. Twelve phages (non-turbid zones of clearing between 3 and 7 mm) were then chosen to study further. The host susceptibility range of isolated phages was determined for the 12 *V. parahaemolyticus* strains (SPA2, SPA3, CHL9, CHL10, BD29, BD44, BD61, BD62, BD63, BD66, BD82, BD84) which comprised our putative phage cocktail (VP10) and further characterization showing protein band profiles (data not shown) indicated 5 distinct biotypes as described in Table 3. DNA fragments were visualized from individual phages used in VP10 and the overall genome size was estimated to be in the range of 21 kb (Figure 7). Restriction fragment length analysis demonstrated that *Eco*RI, *Hin*dIII, and *Hae*III were unable to digest any of the phage isolate DNA at all.

**Table 3:** Characterization of bacteriophage cocktail VP10 and Antibiogram of VP isolates.

**Isolated *Vp* phage/bacteria**	**Vp host source**	**Plaque size (mm)**	**Vp phage profile**	**Major protein band sizes (kDa)**	**Antibiogram**
ΦSPA2	S	6.0	A	112, 96, 93, 64, 55	Gen, Kan, Tet
ΦSPA3	S	5.0	A	112, 96, 93, 64, 55	Tet
ΦCHL9	M	4.0	B	114, 109, 106, 100, 96, 93, 91, 64, 55	Cep, Kan, Tet
ΦCHL10	M	4.0	A	112, 96, 93, 64, 55	Kan
ΦBD29	M	3.0	C	112, 109, 96, 93, 64, 55	Cep, Gen, Kan
ΦBD44	M	4.0	B	114, 109, 106, 100, 96, 93, 91, 64, 55	Cep, Gen, Kan, Tet
ΦBD61	M	6.0	D	106, 103, 102, 99	Cep, Gen, Kan
ΦBD62	M	7.0	A	112, 96, 93, 64, 55	Gen, Kan
ΦBD63	M	7.0	E	112, 109, 104, 98, 90	ND
ΦBD66	M	6.0	A	112, 96, 93, 64, 55	Cep, Van
ΦBD82	M	4.0	A	112, 96, 93, 64, 55	Cep, Gen, Kan
ΦBD84	M	4.0	C	112, 109, 96, 93, 64, 55	Cep, Gen, Kan

*S, seawater; M, mussel (ND: Not determined)*.

### Determination of antibiotic susceptibility of host bacteria

Antibiotic susceptibility/resistance evaluation of bacteriophage host *V. parahaemolyticus* by zone diameter around the antibiotic disks revealed that 82% of the bacteria strains were resistant to kanamycin, 64% were resistant to gentamicin and cephazolin while 36% were resistant to tetracycline. No resistance was observed for ampicillin, chloramphenicol, ciprofloxacin, and vancomycin.

### Activity of phage *in-vitro* against *V. parahaemolyticus*

The results shown in this study indicate that the VP10 cocktail *in vitro* was effective in reducing the numbers of pathogenic *V. parahaemolyticus* in an experimental context. Figure 5 shows some typical results of lytic activity of phages ΦSPA2 and ΦSPA3 against their respective pathogenic *V. parahaemolyticus* hosts at different incubation temperatures. Viable counts of *V. parahaemolyticus* strains SPA2 and SPA3 were reduced by an average of 1 log unit at 30 and 37°C, whereas at 40°C viable counts were reduced by an average of 2 log units. Analysis of variance revealed significant differences (*p* ≤ 0.05) between counts of *V. parahaemolyticus* obtained with the control and treated samples. There was however no significant different between counts (shown in Fig A+B) of *V. parahaemolyticus* SPA2 (*F* = 2.38, *p* = 0.17) and SPA3 (*F* = 1.82, *p* = 0.24) recorded at different temperatures. Multiple comparisons between the effect of phage treatment and temperature of incubation was analyzed using Bonferroni adjustment, and the statistical analysis revealed that there were significance differences (*p* < 0.05) between results obtained at 40°C as compared to 30 and 37°C, there was however no significant difference (*p* = 0.44) between results obtained at 30 and 37°C.

**Figure 4:**
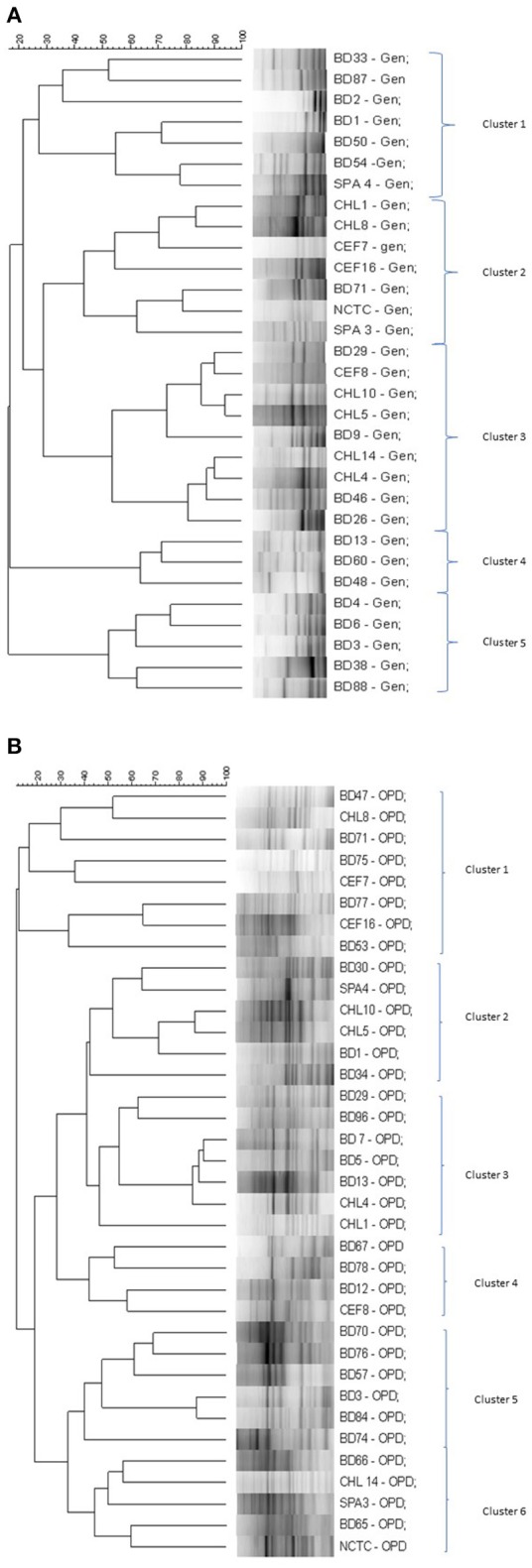
**(A)** Simplified dendrogram based on UPGMA generated by Gel compare software showing genetic similarity from RAPD profiles of *V. parahaemolyticus* isolates (Gen1-50-08). **(B)** Simplified dendrogram based on UPGMA generated by Gel compare software showing genetic similarity from RAPD profiles of *V. parahaemolyticus* isolates (OPD16).

**Figure 5:**
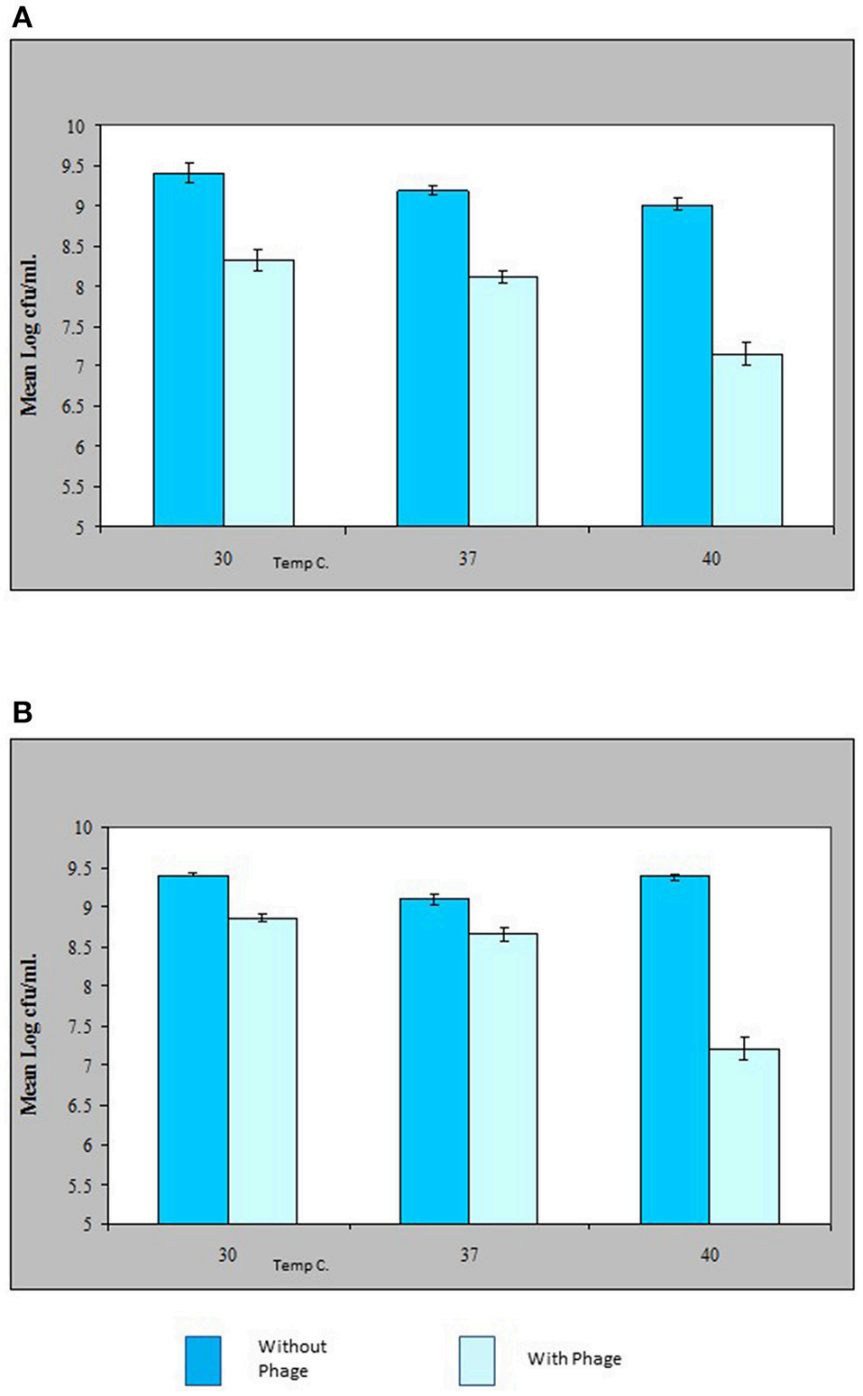
Effect of bacteriophage treatment on *Vibrio parahaemolyticus* SPA2 **(A)** or SPA3 **(B)** in Tryptone Soya Broth incubated at 30, 37, and 40°C. Each bar represents the mean *V. parahaemolyticus* counts of duplicate samples. Error bars denote the standard error of the means of triplicate experiments.

### Activity of phage *in-vivo* against *V. parahaemolyticus*

**Figure 6:**
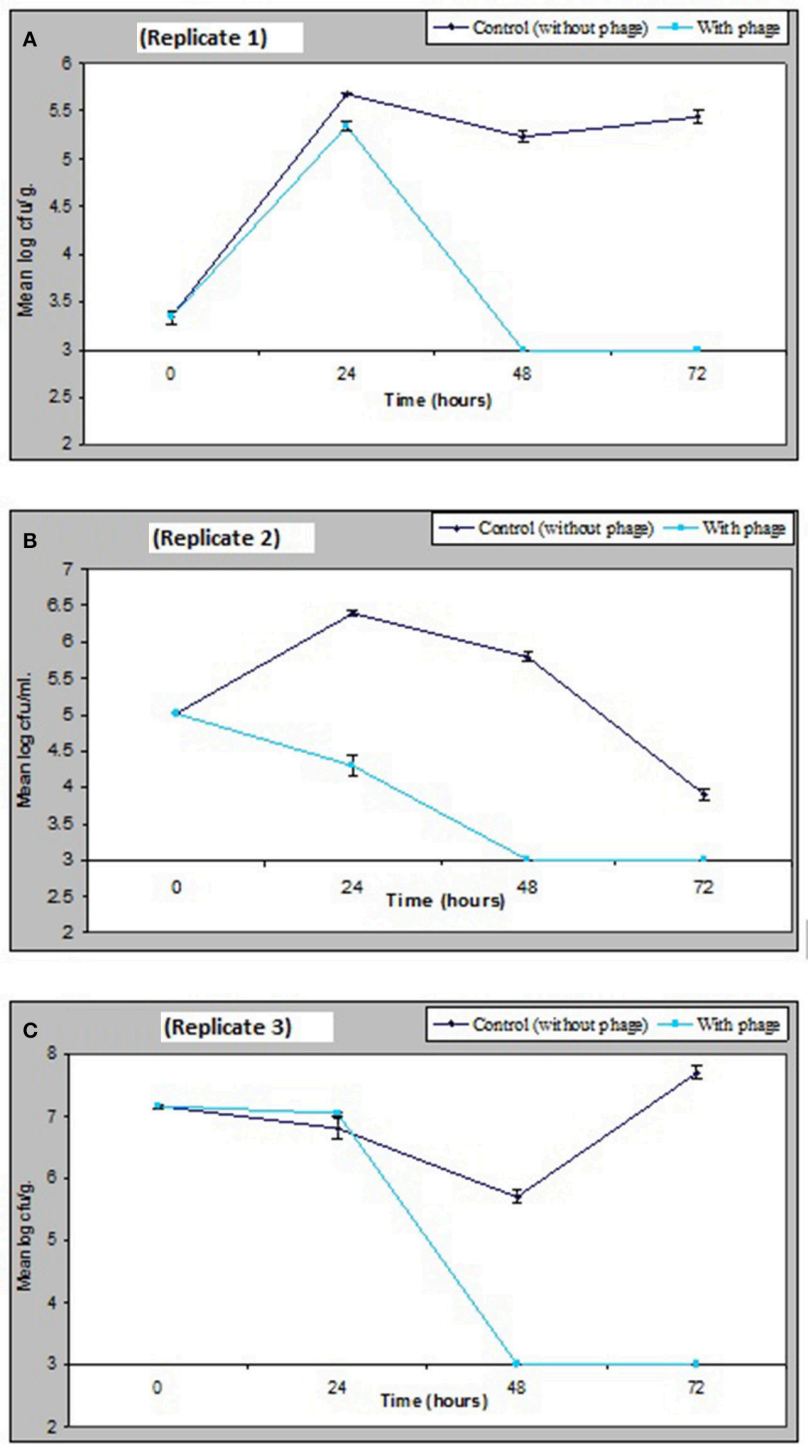
Effect of bacteriophage treatment on *V. parahaemolyticus* in seawater, sediment or mussels after 72 h. Each line represents the mean of duplicate bacteria counts. Error bars denote the standard error of the mean duplicate bacterial counts.

The results obtained in Figure 6 demonstrate that a relatively low numbers of phage in a cocktail has the potential of controlling numbers of *V. parahaemolyticus* impressively *in vivo*. Figure 6 shows that as little as 1.3 × 10^3^ pfu/ml of VP10 phage cocktail was effective in significantly reducing *V. parahaemolyticus* to undetectable numbers in mussels from three replicate experimental tanks after 48 h. *V. parahaemolyticus* was also reduced to undetectable levels after 48 h in seawater and sediment (Figure 6). Analysis of variance revealed significant differences (*p* < 0.01) between counts of *V. parahaemolyticus* obtained in the treated and control (no phage) samples throughout the study period (*F* = 314, *p* = 0.00 for seawater; *F* = 281, *p* = 0.00 for sediment; and *F* = 205, *p* = 0.00 for mussel flesh samples). A lower inoculum of phage cocktail tested 6.75 × 10^2^ pfu/ml produced a similar but less pronounced effect with significant difference between the inoculum size differences for treatments for samples (*F* = 9.9, *p* = 0.04 for sediment and *F* = 37.6, *p* = 0.004 for mussel samples) although not for seawater (*F* = 6.9, *p* = 0.059).

**Figure 7:**
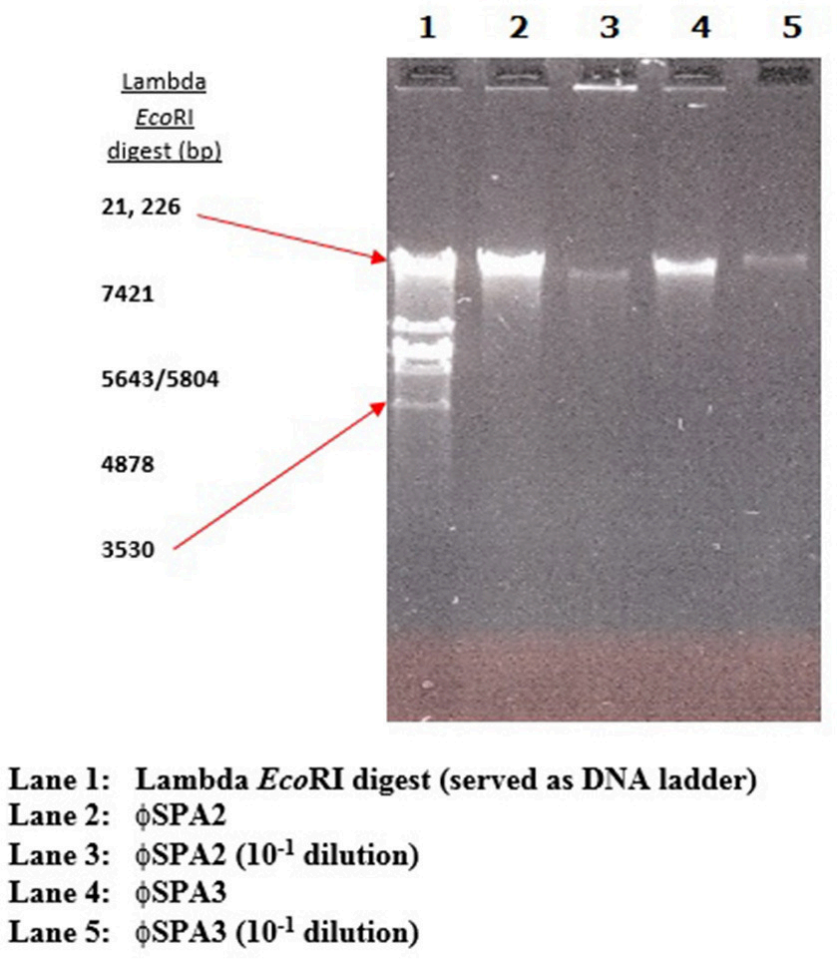
Representative electrophoresis gel (1.0%) of bacteriophage DNA.

## Discussion

CV agar clearly distinguished *V. parahaemolyticus* colonies from other unwanted colonies, confirming the results of other studies (Hara-Kudo et al., 2001; Duan and Su, 2005; Blanco-Abad et al., 2009; Canizalez-Roman et al., 2011). The violet color of *V. parahaemolyticus* remained on CV agar even when they were physically covered by other colored colonies produced by different vibrio species or other bacteria whereas *V. parahaemolyticus* colonies on TCBS agar were occasionally hidden by the yellow color produced by sucrose-fermenting bacteria such as *V. alginolyticus*. The use of CV agar in combination with PCR assay targeting the *tlh* gene was found to be a rapid, sensitive, and accurate method for the isolation and identification of *V. parahaemolyticus* from environmental samples.

The presence of virulence genes *tdh* and *trh* in environmental samples has been reported to be very rare, and in this present study, detection of these pathogenic markers revealed that 2 *V. parahaemolyticus* isolated from retail shellfish samples collected during the preliminary study possessed the *tdh* gene (see Table 2). The low incidence of pathogenic strains (0.9%) in environmental samples (especially retail shellfish) analyzed in this study is interesting when considering the increased risk of human infection associated with them (Bej et al., 1999; Cook et al., 2002; DePaola et al., 2003). The results in this study are however in agreement with studies that have reported only low percentages of environmental isolates possessing *tdh* and/or *trh* (Robert-Pillot et al., 2004; Deepanjali et al., 2005; Canizalez-Roman et al., 2011; Gutierrez West et al., 2013; Letchumanan et al., 2015) and demonstrates that the continued monitoring of environmental *V. parahaemolyticus* is useful and should be encouraged.

*V. parahaemolyticus* are frequently recovered from the water, sediments, suspended particles, plankton, and marine species including shellfish (Joseph et al., 1982; Daramola et al., 2009; Zhang and Orth, 2013). As reported by Kaneko and Colwell (1975), El-Sahn et al. (1982), Ristori et al. (2007), and Martinez-Urtaza et al. (2008) the numbers of *V. parahaemolyticus* in the environment and in seafood has been reported to vary greatly according to season, location, sample type, and analytical methodology employed. Figure 1A shows the study location of the present surveillance study and Figures 2A–C present *V. parahaemolyticus* counts in seawater, sediment, and mussels, respectively. Although another study by Cook et al. (2002) revealed significant correlation between temperature and level of *V. parahaemolyticus* in shellfish, others have reported that there was no correlation between temperature and salinity and numbers of *V. parahaemolyticus* (Kaneko and Colwell, 1973; Thompson and Vanderzant, 1976; Deepanjali et al., 2005; Ristori et al., 2007). In this present study we found no significant correlation between level of *V. parahaemolyticus* (in seawater and sediment) and temperature and salinity. Similar result were also observed for level of *V. parahaemolyticus* in mussels and seawater and salinity. For mussel samples *V. parahaemolyticus* were detected in all mussel samples throughout the study period and the counts appeared to be independent of variation recorded in salinity and temperature. The environmental drivers that affect the population of *V. parahaemolyticus* in shellfish are complex (Paranjpye et al., 2015) as other factors beyond temperature and salinity have been shown to also play a part. Factors such as nutrients, chlorophyll-a, plankton, dissolved organic carbon and turbidity have varying effects depending on species, geographic location, or environmental niche (Takemura et al., 2014). Although in this study our focus was mainly on observing the effect of seawater and sediment temperature and salinity on level of *V. parahaemolyticus*, other factors such as temperature of mussel meat, level of nutrients present in the environment were not considered. Further studies incorporating additional variables including internal temperature of mussel meat might provide additional insight into the conditions that impact levels of *V. parahaemolyticus* in environmental samples.

The prevalence of *V. parahaemolyticus* in marine animals during cold and warm months and the lack of seasonal variation in numbers of environmental *V. parahaemolyticus* in temperate zones has been reported by Thompson and Vanderzant (1976). Lack of recent comparative data is surprising and our results clearly suggest the continual presence of *V. parahaemolyticus* in mussels in the estuarine environment of the temperate River Humber throughout the year.

In the RAPD study, all the clusters generated by the two primers suggest inter-clustering of the “clinical” and “environmental” isolates except clusters 4 and 5 in Figure 4A and cluster 5 in Figure 4B which were observed to group together isolates from the study area. Isolates of *V. parahaemolyticus* have previously been shown to be heterogeneous by RAPD-PCR from a variety of geographical locations and climates (Sudheesh et al., 2002; Bilung et al., 2005; Ellingsen et al., 2008; Sahilah et al., 2013). Our study confirms a high degree of genetic diversity and strain variation within both “clinical” and “environmental” categories.

Viruses infecting bacteria are an essential biological component in aquatic microbial food webs. They play a key role in controlling cell mortality, nutrient cycles, and microbial diversification for planktonic communities (Jacquet et al., 2010). Phages are similar to antibiotics in that they have remarkable killing activity. All bacteriophages known to date are specific, reacting to only their targeted bacterial host and not to human or other eukaryotic cells. This is in clear contrast to broad spectrum antibiotics which target both pathogenic microorganisms and the normal microbiota. Phages are generally isolated from environments that are habitats for their respective host bacteria (Vinod et al., 2005). In this present study, all the samples from which *V. parahaemolyticus* has been isolated (seawater, sediment, and shellfish) were tested for the presence of bacteriophage against *V. parahaemolyticus* and seawater and mussel samples tested positive for the presence of these phages which suggests that marine samples being the habitat for *V. parahaemolyticus* would be ideal source for isolation of *V. parahaemolyticus* phages.

The investigation of *V. parahaemolyticus* phage distribution in the marine environment using cultures of bacterial hosts combined with plaque or lysis assay, shows that phages are ubiquitous and present in varied numbers. However, the isolation of phages from the estuarine environment was not without difficulty. Repeated attempts were carried out before successfully isolating these phages, also after successfully isolating phages it was observed that mussel samples yielded more *V. parahaemolyticus* specific phage as compared to seawater, this observation is in agreement with Baross et al. (1978) who reported higher incidence of bacteriophage in marine animals as compared to sediment and seawater. Reports of difficulty in isolating bacteriophage from marine samples other than shellfish have also be published by Johnson (1968) who investigated marine mud samples for presence of bacteriophages but was successful only once out of 15 attempts. Also, Hidaka and Tokushige (1978) sampled 18 enriched seawater samples from different locations but found phages for only 11 isolates of *V. parahaemolyticus*. Hidaka (1973) isolated 165 bacteria from 6 marine locations and obtained only 16 phage-host systems. Remarkably, Baross et al. (1978), also obtained phages from seawater from only two out of 64 trials, and from sediments in four out of 69 attempts. Although further research is required to determine incidence of phages in different marine samples, Baross et al. (1978) however suggests that higher frequency in the isolations of bacteriophages from marine animals might be an indication that these viruses play an important role in the ecology of marine vibrios.

In this present study, isolated bacteriophages produced distinctive plaque morphologies when plated on lawns of their host bacterium using soft-agar overlay technique. Both turbid and clear plaques were isolated and based on the morphology of the plaques produces by the bacteriophages, 12 out of the 61 isolated phages were selected because they produced very clear plaques on lawns of host bacteria which strongly suggested that they were lytic (Jurczak-Kurek et al., 2016).

Molecular characterization of the phages by restriction fragment length analysis of bacteriophage DNA revealed that *Eco*RI, *Hind*III, and *Hae*III were unable to digest the phage DNA. This observation is in agreement with that of Sen and Ghosh (2005), they have also found resistance to *Eco*RI in marine bacteriophage of *V. cholerae*. Also Dutta and Ghosh (2007) observed that vibrio bacteriophage Phage S20 DNA was found to be resistant to *Eco*RI and other restriction enzymes. Bacteriophage defense adaptations to avoid host restriction systems might be an explanation to the observed restriction enzyme resistance of isolated phage in this study. Among these defense adaptations are the unusual modification of DNA, low frequency of target sequences, and the production of a protein that interferes with one or more of the activities of a Restriction-Modification system (Murray, 2000).

The use of protein analysis to characterize bacteriophage proved to be a straightforward and useful tool in characterizing phages as compared to PCR-RFLP, the band profile was able to reveal 5 patterns. The use of SDS-PAGE has been employed by other researchers and they have also found the method to be useful in characterizing vibrio bacteriophages (Alonso et al., 2002; Walter and Baker, 2003; Dutta and Ghosh, 2007).

Antibiotic treatment is necessary for controlling *V. parahaemolyticus* infections however the overuse of antibiotics has brought about antimicrobial resistant bacteria, which is becoming a major concern for human health (Ji et al., 2011; Shaw et al., 2014; Blair et al., 2015; Letchumanan et al., 2015). The overall resistance pattern of the phage host *V. parahaemolyticus* investigated in this study reveals resistance to cephalozin (64%) which is in agreement with Li et al. (2017), gentamicin (64%), kanamycin (82%), and tetracycline (36%). No resistance was however recorded for ampicillin, chloramphenicol, ciprofloxacin, and vancomycin. The result obtained for ampicillin and chloramphenicol is agreement with the data published by Letchumanan et al. (2015). Letchumanan et al. (2015) reported that only 4 and 5% of strains tested were resistant to ampicillin and chloramphenicol respectively. This is however contrary to the result obtained by Li et al. (2017) especially for ampicillin for which 100% resistance was reported for strains tested in their study. Foods contaminated with antibiotic resistant determinants may be transferred to other bacteria of clinical significance; and *V. parahaemolyticus* is a candidate vehicle for such transfer because of its diversity and its ability survive in the gastrointestinal tracts of both human and animals (Zulkifli et al., 2009).

The results obtained from this present research confirms the role of the environment and most especially contaminated shellfish as a reservoir of antibiotic resistant bacteria some of which contain antibiotic resistance genes which can easily be transferred to other bacteria of clinical significance and so causing a major problem for humans and a threat to public health.

Bacteriophages are natural and most abundant biological entities in the environment, it is estimated that there are about 10^31^ phages on earth and they are ~10 times more than their bacterial hosts (Abedon et al., 2011; Burrowes et al., 2011). The potential effect of specific bacteriophage in controlling the population of their target host bacteria has been studied extensively and quantified over the past decades however to the best of our knowledge the impact of bacteriophages already present in the environment on marine bacterial population has not been quantitatively measured. Although the effect of viruses on the populations of microbes that co-exist with them in the same environment is unknown and has not been quantified in this study however, the fact that vibrio phages could not be isolated without previously propagating in enrichment medium indicates that densities of specific vibrio phages (able to infect our isolated and reference strains) are relatively low in the marine samples tested. Also some samples from which *V. parahaemolyticus* were isolated did not harbor their corresponding phages, suggesting that specific hosts and lytic phages did not always coexist in the same environment (Tan et al., 2014).

The majority of all marine phages are highly host specific (Coetzee, 1987) and 75% lyse only the original host bacterium. One of the phages (ΦBD61) in our study formed clear plaques on 2 other *V. parahaemolyticus* isolates (BD62 and BD63), but was unable to form plaques on lawns of 9 of our *V. parahaemolyticus* isolates. This result is in agreement with Sklarow et al. (1973) who showed that *V. parahaemolyticus* phages are highly specific; giving no lytic response on 53 *V parahaemolyticus* strains or 95 other *Vibrio* strains in their study. For effective phage applications it is probably necessary to design highly multi-component cocktails as demonstrated here. Also for identification and selection of phages for phage therapy the use the efficiency of plating (EOP) method can enhance the selection of rather than the spot test (Mirzaei and Nilsson, 2015) which reflects the bactericidal effect of phages.

Studies into the potential of bacteriophage as agents of therapy against bacterial pathogens in aquaculture have been reviewed (Nakai and Park, 2002; Jun et al., 2013; Madsen et al., 2013). In the present study, in comparison with controls remarkably small inocula tested significantly reduced the recovery from naturally contaminated mussels and seawater. Temperature, pH, and salinity play important roles in the ability of phage-control of bacterial pathogens. Phage are generally more stable in alkaline pH than in acidic pH, and vibriophage are stable at pH range of 5.0–9.0 (Dutta and Ghosh, 2007). Lower pH can interfere with lysozyme or protein coats preventing phage attachment to receptor sites of the host cell (Leverentz et al., 2004). Vibriophage can be sensitive to elevated temperatures interfering with phage attachment, and *V. parahaemolyticus* phages have a requirement for salts at marine concentrations for infection and growth. However, very high salt concentrations can adversely affect the protein coat and phage enzyme activity. At low salt concentrations, salt ions interact with proteins and stabilize protein structure by neutralizing protein charges (Baross et al., 1978; Fennema, 1996).

Phage biocontrol of foodborne pathogens is an interesting idea, which is gaining momentum as a feasible alternative to antibiotic use. Several studies describing such uses have now been published and reviewed. These include the application of phage to control *Salmonella* species in bean sprouts (Ye et al., 2010), broiler carcasses (Fiorentin et al., 2005; Atterbury et al., 2007), frankfurters (Guenther et al., 2012), chicken skin (Hungaro et al., 2013), cheese (Modi et al., 2001), and melon (Leverentz et al., 2001); *Listeria monocytogenes* on melon (Leverentz et al., 2003, 2004; Hong et al., 2015) and cheeses (Guenther and Loessner, 2011); *Campylobacter* on chicken skin (Atterbury et al., 2003); and *Escherichia coli* O157 on retail beef (Wang et al., 2017). Commercial production of the first phage for use in foods was in the Netherlands, with the Listex™ P100 product (Carlton et al., 2005) which was launched to control *Listeria* in cheese and meat. This preparation received FDA approval.

Considerations in designing phage biocontrol strategies must include pH, temperature, and salt concentration in the microenvironment although the possible response of the host defense in shellfish and physiology of the pathogen *in-vivo* may be equally important. A more complete understanding of the environmental and physiological influences should provide some explanation for the disparity among phage lytic activities *in vitro* and *in vivo*, and enable the design of the more effective phage biocontrol strategies in the future. Another important impediment to phage biocontrol is the requirement for a threshold density of bacterial host cells. The need for bacterial populations of 3 to 5 log cfu/ml has been reported for phage to have an impact upon hosts (Wiggins and Alexander, 1985; Kennedy and Bitton, 1987).

In phage therapy a key determinant of the phage potential to eradicate bacterial targets is phage density in relation to the host cells. Multiplicity of infection (MOI) is the ratio between the number of viruses in an infection and the number of host cells and can be determined by adjusting the relative concentration of virus and host (Thomas, 2001). It is important to be able to adjust these concentration of virus and host and calculate phage dose (phage density) required for effective phage therapy (Thomas, 2001; Abedon et al., 2011). However, defining MOI in terms of the number of phages that have to be added to bacteria can be misleading especially so when densities of bacteria are low or phage adsorption slow (Abedon, 2016). The use of MOI as the sole means of describing dosing during phage-mediated biocontrol of bacteria (Payne and Jansen, 2001; Harper, 2006; Abedon, 2009) can be a problem during phage therapy. The simple ratio of virus particles to cells given by the MOI is also shown by Kasman et al. (2002), to be illogical in the absence of information about other parameters such as the density of host cells, the adsorption constant of the phage, the number of phage, and the length of time for which they interact.

In our *in vitro* and *in vivo* studies, the levels of *V. parahaemolyticus* at the point of introduction of the phages were 10^3^ and 10^9^ cfu/ml respectively, from these studies it seems that number of bacteria (whether low or high) are unlikely to affect the effectiveness of phage in bacteriophage therapy. This result shows the potential of the use phages in systems where low level of bacterial pathogens are found (i.e. in naturally contaminated food which can sometimes have as low as 1 × 10 CFU/g or ml of pathogen present). As discussed by Hagens and Loessner (2010), in as much as the concentration of the reaction partners (phage) is sufficient enough to enable contact and subsequent reaction (infection and killing) the other reaction partner can be present at a very low Concentration (numbers of bacteria). The researchers state that, in fact, once a critical concentration threshold of phage numbers is reached to enable it to cover the entire available space within any given matrix, the concentration of the bacterial host is not important, i.e., it does not matter whether only 1 or 10^6^ cells per ml are present, they will all be infected (Hagens and Loessner, 2010).

Another problem with phage therapy is the narrow host specificity of bacteriophages, in a study conducted to control food spoilage organisms it was shown that if the host range of a bacteriophage is too narrow biocontrol is ineffective (Greer and Dilts, 1990). For effective biocontrol therefore, phages should have a well-balanced/almost perfect host range which is neither too narrow nor too broad. An example of such phage is Felix O1 which lyses 96–99.5% of *Salmonella* serovars (Lindberg, 1967). Also for successful biocontrol the use of phage cocktail (rather than a single phage) is required and this approach has proved successful in a study published by O'Flynn et al. (2004) who investigated the use of phages for controlling *Escherichia coli* O157:H7 on beef.

Finally, another obstacle to phage therapy is public perception, would the average consumer respond favorably to the introduction of viruses to their food? The proof of the safety of phage have been demonstrated by phage therapy pioneers who deliberately ingested the phage preparations themselves. In 1919, d'Herelle, Hutinel, and several hospital interns ingested a phage preparation which was intended to treat dysentery before administering it to a 12-year old boy in order to confirm the safety of the phage preparation (Summers, 1999). Bruttin and Brussow (2005) also reported the safe intake of T4 phage by volunteers. In addition in an American article it was indicated that some people see phages as “green” and environmentally friendly (Fox, 2005). Phages have also been seen as a “natural” alternative to chemical preservatives. Whatever the perceptions concerning safety of phages it is a fact that phages are globally numerous (10^31^) and a natural component of foods (Tsuei et al., 2007) which are being ingested by everyone every day. The direct application of phages to food or food processing protocols is only one of many biocontrol approaches that might be adopted.

## Conclusion

The results obtained in this study reveal the occurrence of *V. parahaemolyticus* in the temperate conditions of the River Humber throughout the year. In particular *V. parahaemolyticus* contaminated mussels were recovered from the temperate estuary throughout the colder months in contrast to other studies. Uncooked or partly cooked non-farmed shellfish consumption presents increased risk from temperate estuarine harvests at any time of the year. No distinct clustering occurred following RAPD-PCR analysis between environmental and “clinical” isolates. Specific cocktails of bacteriophages tested against recovered isolates of *V. parahaemolyticus* in experimental settings significantly reduced the organism *in vitro* and *in vivo*. Our preliminary work reported here shows that potential eradication *in-vitro* and *in vivo* by specific cocktails of phage in low numbers in an experimental setting provides evidence of the valuable potential of phage as a decontamination agent in mussel processing.

## Author contributions

BO designed and performed all the experiments and associated data analysis and prepared a draft manuscript. BO also participated in the coordination of the project. RD conceived the study, designed and coordinated the project. RD co-authored the manuscript revising it for important intellectual content. Both authors read and approved the final manuscript.

### Conflict of interest statement

The authors declare that the research was conducted in the absence of any commercial or financial relationships that could be construed as a potential conflict of interest.
